# Outcome of brain metastases from adrenocortical carcinoma: a pooled analysis

**DOI:** 10.1007/s40618-023-02140-1

**Published:** 2023-06-24

**Authors:** A. Turla, M. Laganà, V. Cremaschi, M. Zamparini, L. De Maria, F. Consoli, A. Abate, M. Tamburello, A. Alberti, S. Sigala, S. Grisanti, M. M. Fontanella, D. Cosentini, A. Berruti

**Affiliations:** 1 Medical Oncology Unit, ASST Spedali Civili di Brescia, Department of Medical and Surgical Specialties, Radiological Sciences, and Public Health, University of Brescia, Brescia, Italy; 2 Neurosurgery Unit, Department of Medical and Surgical Specialties, Radiological Sciences and Public Health, Spedali Civili di Brescia, University of Brescia, Brescia, Italy; 3 Section of Pharmacology, Department of Molecular and Translational Medicine, University of Brescia, Brescia, Italy

**Keywords:** Adrenocortical carcinoma, Cerebral metastases, Endocrine tumors, Pooled analysis

## Abstract

**Purpose:**

Brain metastases rarely complicate the natural history of patients with adrenocortical carcinoma (ACC). No information is available regarding the life expectancy and efficacy of treatments in ACC patients with brain involvement.

**Methods:**

A pooled analysis was performed by searching on PubMed and using the keywords: “brain metastases in adrenocortical carcinoma”, and “leptomeningeal metastases in adrenocortical carcinoma”. Four patients diagnosed at Spedali Civili Hospital in Brescia were added to the analysis. Data concerning demographic, disease characteristics, adopted treatments and patient prognosis were collected.

**Results:**

A total of 27 patients (18 adults and 9 children) were included in this study, 22 of them had an adequate follow-up. Brain metastases occurred late in the natural history of adult patients but not in that of children. Surgery plus/minus radiation therapy was the treatment of choice. Adult patients with brain metastases had a poor prognosis with a median progression-free survival (PFS) and overall survival (OS) of 2 and 7 months, respectively. Median PFS and OS were not attained in children.

**Conclusion:**

Brain metastases in ACC patients are rare and are associated with poor prognosis, particularly in adults. Surgery plus/minus radiotherapy is the only therapeutic approach that can offer patients a chance to obtain durable local disease control.

**Supplementary Information:**

The online version contains supplementary material available at 10.1007/s40618-023-02140-1.

## Introduction

Adrenocortical carcinoma (ACC) is a rare malignancy affecting around 0.7–2 persons per one million population per year. [1, 2] The incidence shows a bimodal distribution with the first peak in childhood (1–6 years old) and the second peak in adults between 46 and 55 years. Surgery, with or without adjuvant mitotane therapy [3], is the reference treatment in ACC patients with early disease stage [4], however, only 50% of patients at diagnosis have an operable disease. Mitotane either administered alone [5] or in combination with the EDP regimen (etoposide, doxorubicin, and cisplatin) [6, 7], represents the current standard systemic therapy for ACC patients with locally advanced or metastatic disease, not eligible for surgery. Systemic antineoplastic therapy has limited efficacy and the prognosis of ACC patients with metastatic disease is usually poor with an expected 5-year survival of approximately 15% [1, 2]. Mitotane plus/minus EDP is the standard therapy also in the management of children, although the protocols for the use of systemic therapy in children are not comparable to that of adults [8] and the prognosis of ACC in childhood is generally better than that in adulthood [9]. The European Network for the Study of Adrenal Tumours (ENSAT) staging system [10] with a modified version for metastatic patients (mENSAT) [11], the proliferation activity assessed by ki67 immunohistochemistry [12] and cortisol hypersecretion [13] are widely used as prognostic factors. To improve prognostication [14], the S-GRAS score (tumor Stage—Grade, R status, Age and Symptoms) was introduced in clinical practice, combining the different clinical and histopathological parameters associated with prognosis [15]. Furthermore, the S-GRAS score was adapted to the pediatric population creating the pediatric scoring system pS-GRAS [16].

The most common metastasis sites in ACC patients are, in order of frequency, regional lymph nodes, lungs, and liver [11]. Bone involvement is relatively rare, but a cause of morbidity [17]. Conversely, intracranial metastases are rare: only few cases have been described in the literature and the prognostic impact of brain involvement has not been demonstrated in ACC patients. In this study, we performed a pooled analysis of all published cases in both adult and pediatric ages to obtain information on the onset of brain metastases, treatment strategies, and prognosis. We also included in the database four ACC patients followed in our Institution developing brain and leptomeningeal metastases.

## Results

### Case series

From 2012 to 2021, 225 consecutive patients were treated and followed at the Medical Oncology Unit of the ASST-Spedali Civili in Brescia, University of Brescia (Italy). Four of them (1.8%) developed brain metastases. These cases are described below; their characteristics are summarized in Supplementary Table 1.

#### Case 1

A 58-year-old male patient with stage IV ACC (primary disease and lung metastases) was referred to our Center in April 2015. The endocrinological profile performed did not reveal hormonal hypersecretion. The patient was initially treated with 6 cycles of EDP-M obtaining a partial response of primary ACC and a complete response of the lung metastases. In September 2015 the patients underwent surgery and became disease-free. Mitotane therapy was continued.

In July 2018 a CT scan revealed pulmonary recurrence and in August 2018 cabazitaxel therapy was proposed in the CABACC trial [18]. At the same time, the patient complained of aphasia, right hemiparesis, and cognitive-motor slowing. Brain MRI showed an intra-axial lesion in the frontal zone of the left hemisphere, measuring about 2.2 cm in diameter, with abundant vasogenic edema surrounding the lesion and compressing the lateral left ventricle with a midline shift to the right (Supplementary Fig. 1).

In August 2018 the patient underwent radical surgery and became macroscopically free of disease (Supplementary Fig. 1). The neurological deficits almost completely regressed. Histology revealed metastatic localization of ACC. Cabazitaxel therapy started in October 2018.

In November 2018 an MRI revealed brain recurrence (Supplementary Fig. 1) and the patient was addressed to panencefalic radiotherapy. Cabazitaxel was continued till March 2019 due to clinical benefit. From that date onwards the patient’s clinical conditions rapidly worsened leading to death in April 2019.

#### Case 2

A 63-year-old male patient was diagnosed with ACC in May 2017 when an abdominal CT scan revealed a left adrenal mass of 15 cm, which was endocrinologically silent (ENSAT stage II). The disease was surgically removed in June 2017. No adjuvant therapy was prescribed since the patient refused any post-operative treatment. During the postoperative follow-up, bilateral pulmonary and hepatic nodules were found in January 2019, so he was addressed to chemotherapy with the EDP-M scheme. In October 2019 a chest-abdomen CT scan documented a disease progression with pulmonary, hepatic, and peritoneal metastases. In the same period, due to the occurrence of neurological symptomatology, a brain CT showed an extra-axial epidural right frontal lesion with involvement of the cranial theca and adjacent soft tissues. A brain MRI confirmed the right frontal expansive lesion, without the involvement of the meningeal plane.

After a multidisciplinary discussion, carefully evaluating the risk/benefit ratio, we decided to address the patient to surgery and in November 2019 the brain mass was removed with the aid of neuronavigation. The definitive histological examination confirmed the diagnosis of ACC metastasis and the surgical margins appeared microscopically clean.

The postoperative course was characterized by the absence of general clinical and neurological focal complications. The patients died in May 2020 due to extra-brain disease progression.

#### Case 3

A 55-year-old woman underwent an abdominal ultrasound in 2018 due to the onset of abdominal pain. A left adrenal mass of 12 cm was diagnosed. A staging CT scan confirmed the adrenal lesion and revealed the presence of 3 lung metastases. The endocrine work-up was negative for hormone hypersecretion. A biopsy of the abdominal mass was performed in another center, revealing neoplastic cells of epithelial origin characterized by moderate nuclear pleomorphism, negative at the immunohistochemistry for PAX-8, TTF-1, and chromogranin, positive for cytokeratin AE1/AE3, cytokeratin 7, calretinin, inhibin, synaptophysin, and melan A. These features were compatible with the diagnosis of primary ACC, so the patient was addressed to the Medical Oncology Unit at Spedali Civili as a reference center. In August 2018 the patient underwent neoadjuvant chemotherapy with the EDP schedule, associated with oral mitotane. A CT scan performed after 6 cycles showed a partial response of the primary ACC and a complete response of lung metastases. Chemotherapy was interrupted and the patient was maintained on mitotane. In February 2019 a left nephrosurrenalectomy was performed, followed by hyperthermic intraperitoneal chemotherapy (HIPEC).

A CT scan performed in December 2019 revealed lung disease progression and a second-line treatment with Gemcitabine-Capecitabine was administered. In April 2020 a disease progression was documented with the appearance of multiple hepatic nodules. A rechallenge with cisplatin was then administered, but in November 2020 the patient was hospitalized due to the appearance of left brachio-crural hemiparesis. A brain CT scan found an intra-axial right parietal expansive lesion, measuring 41 mm and surrounded by abundant vasogenic edema; the lesion caused left midline shift and right transtentorial uncal herniation (Supplementary Fig. 2).

No symptomatic/palliative radiotherapy was feasible due to the high risk of severe, potentially fatal, neurologic complications. High-dose steroid therapy was prescribed to reduce the edema and domiciliary palliative care assistance was organized; the patient died at the end of November 2020.

#### Case 4

Due to weight loss, a 42-year-old male patient performed an abdominal ultrasound in October 2017 which revealed a right adrenal mass. A CT scan showed an 11.5 cm adrenal mass without metastases; no pathological hormonal secretion was found at laboratory work-up. An adrenal biopsy that documented morphological and immunohistochemical characteristics of ACC (immunohistochemical positivity for melan A and SF-1) was carried out in another center; for this diagnosis, the patient was then referred to Spedali Civili in Brescia. After a careful multidisciplinary discussion, he was not immediately addressed to surgery but to neoadjuvant treatment with the EDP-M scheme. In December 2017 the first cycle of chemotherapy was administered, being well tolerated. However, during the hospitalization, the patient suddenly suffered from an epilepsy attack, so a brain CT scan was urgently performed revealing suspected meningitis of unclear origin. A neurologic evaluation, a brain MRI (Supplementary Fig. 3), and an electroencephalogram were performed, but they did not attain a definitive diagnosis.

A rachicentesis was then performed and the analysis of cerebrospinal fluid revealed the presence of ACC cells. Due to the leptomeningeal diffusion and the rapid worsening of the patient’s performance status, chemotherapy was interrupted and symptomatic therapy with high-dose steroids and antiepileptics was introduced. The patient was then addressed to palliative care assistance in hospice and died a few weeks later in January 2018.

### Literature search results

**Fig. 1:**
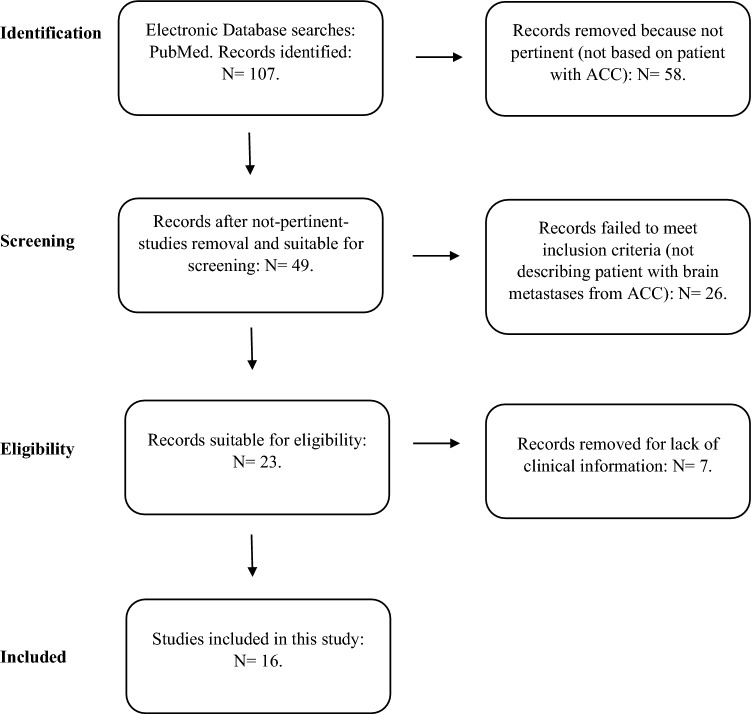
PRISMA flow chart diagram: literature selection process

From one hundred and seven records identified using the previous keywords, fifty-eight papers were removed because not pertinent, twenty-six for failing the inclusion criteria. Seven additional records were not included in the analysis for lack of clinical information (Fig. 1).

Thus, sixteen articles were selected [19–34] for a total of twenty-three patients (fourteen adults and nine children) to whom the four cases followed at Spedali Civili in Brescia were added.

### Patients’ characteristics

Data from eighteen adults and nine pediatric patients were collected, and their characteristics are shown in Tables 1A and 2A, respectively.

**Table 1:** (A) Patients’ characteristics of the adult population, (B) Clinical presentation and treatments of brain metastases in the adult population

Characteristics	Value
(A)	
Patients included in the analysis	18
Median age (years, range)	44.5 (23–63)
Gender (%)	
Male	10 (56)
Female	8 (44)
Symptoms at diagnosis (%)	
Cushing’s Syndrome	5 (28)
Pain	6 (33)
Asymptomatic	7 (39)
ENSAT stage at diagnosis	
I–II	7 (39)
III	4 (22)
IV	7 (39)
(B)	
Number of CNS metastases^a^ (%)	
Single	9 (75)
Two	2 (17)
Three	1 (8)
Localization of CNS metastases (%)	
Brain	13 (72)
Meninges	3 (17)
Both	2 (11)
Concomitant localizations (%)	
Cerebellum	2 (11)
Ocular	2 (11)
Brain lobe^a^ (%)	
Frontal	6 (46)
Parietal	6 (46)
Temporal	0 (0)
Occipital	1 (8)
Side^a^ (%)	
Left	3 (25)
Right	9 (75)
Presentation at CT/MRI (%)	
Mass without perilesional edema	5 (28)
Mass with perilesional edema	5 (28)
Intralesional hemorrhage	3 (16)
Meningeal carcinomatosis/meningeal involvement	5 (28)
Symptoms related to brain metastases^a^ (%)	
Hemiparesis	7 (41)
Postural instability	2 (11)
Seizures	3 (18)
Headache	1 (6)
Diplopia/ptosis	1 (6)
Nausea, vomiting and lethary	1 (6)
Aphasia	1 (6)
Asymptomatic	1 (6)
Treatment for brain metastases^a^ (%)	
Surgical exeresis	6 (35)
Radiotherapy (Gamma Knife)	3 (17)
Surgical exeresis followed by adjuvant radiotherapy	4 (24)
Symptomatic therapy (steroids, antiepiletics)	4 (24)

*CNS* central nervous system

^a^The common denominator of the proportions refers to the number of patients for whom the data is available

**Table 2:** (A) Patients’ characteristics of the pediatric population, (B) Clinical presentation and treatments of brain metastases in the pediatric population

Characteristics	Value
(A)	
Patients included in the analysis	9
Median age (years, range)	9 (1 day-14 years)
Gender (%)	
Male	5 (56)
Female	4 (44)
Symptoms at diagnosis^a^ (%)	
Cushing’s Syndrome + sex hormone secretion	3 (60)
Precocious puberty alone	1 (20)
Bradycardia and skin lesions	1 (20)
ENSAT stage at diagnosis^a^	
I–II	3 (60)
III	0
IV	2 (40)
(B)	
Number of CNS metastases^a^ (%)	
Single	3 (75)
Multiple	1 (25)
Localization of CNS metastases (%)	
Brain	8 (89)
Meninges	1 (11)
Brain lobe^a^ (%)	
Parietal	1 (20)
Parietal-frontal	1 (20)
Lateral ventricle	2 (40)
Cerebellum	1 (20)
Side^a^ (%)	
Left	5 (71)
Right	2 (29)
Presentation at CT/MRI^a^ (%)	
Mass without perilesional edema	3 (50)
Mass with perilesional edema	2 (33)
Meningeal carcinomatosis/meningeal involvement	1 (17)
Symptoms related to brain metastases^a^ (%)	
Diplopia/ptosis/blurred vision	2 (40)
Difficulty in naming and repetition	1 (20)
Signs of increased intracranial pressure	1 (20)
Asymptomatic	1 (20)
Treatment for brain metastases^a^ (%)	
Surgical exeresis	4 (80)
Radiotherapy (Gamma Knife)	0 (0)
Spontaneous resolution	1 (20)

*CNS* central nervous system

^a^The common denominator of the proportions refers to the number of patients for whom the data is available

The median age of the adult population was 44.5 years (23–63) and the majority were males (ten patients, 56%). Five patients (28%) presented Cushing’s syndrome. Fourteen patients (78%) had their primary ACC completely removed. Adjuvant therapy was prescribed in six patients (33%) consisting of mitotane monotherapy in four cases and mitotane with chemotherapy in the remaining two. Five patients (28%) had metastatic disease at diagnosis.

In the pediatric population, the median age was 9 years (1 day-14 years) with a prevalence of males (five patients, 56%). In the five records in which the information was available, three patients (60%) had symptoms of Cushing’s Syndrome combined with sex hormonal secretion, while in one case (20%) precocious puberty was the only presentation of the disease.

The remaining child had no clinical signs of hormonal production, despite serum levels of DHEA sulfate and 17-hydroxyprogesterone consistently elevated. [28].

The presence of Beckwith-Wiedemann syndrome was reported in one patient. [33] Hemihypertrophy, suggestive of Beckwith-Wiedemann syndrome, was described in additional two patients [28, 32], while in another patient there was a suspect of Li Fraumeni syndrome [34]. Five patients (83%) had complete resection of primary ACC and none of them underwent adjuvant therapy.

In one patient the disease showed a peculiar clinical history. ACC diagnosis was performed at birth with brain (right frontal and left parietal lobes) and skin metastases. On the 22nd day of life, the patient underwent surgery for ACC and a wedge liver biopsy, that excluded liver metastases. The patient was then observed closely, and no systemic therapy was introduced. Partial regression of the cutaneous nodules was noticed after 2 months from surgery, and all skin lesions resolved spontaneously after 4 months. Brain metastases also completely disappeared at MRI performed after 4 months from surgery. The patient was alive and disease free one year after diagnosis.

### Brain/leptomeningeal metastases: presentation and treatment

Tables 1B and 2B show the details related to brain metastases and their clinical approach in adult and pediatric patients, respectively; all the percentages refer to the number of patients for whom data were available.

In the adult population (Table 1B) brain/leptomeningeal metastases occurred at a median time interval of 26.5 (2–132) months from the diagnosis, while the first diagnosis of metastatic disease occurred at a median time of 4 months from the diagnosis of ACC (0–107). Brain metastases were single in nine cases, and multiple in three (two lesions in two patients, three in one patient), whereas this information was not available for six patients. Thirteen patients (72%) had encephalic metastases, three (17%) meningeal ones, while two patients (11%) reported lesions spreading in both sites. The most involved lobes were frontal (six patients, 46%), parietal (six patients, 46%), and occipital (one patient, 8%). Two patients had cerebellar involvement and two ocular metastases. The most frequent neurological symptoms were hemiparesis (seven patients, 41%), seizure (three patients, 18%), and postural instability (two patients, 11%). In one case the patient was asymptomatic at the diagnosis. Less commonly there were headaches (one patient, 6%); diplopia and palpebral ptosis (one patient, 6%); lethargy, weakness, nausea, vomiting (one patient, 6%); aphasia (one patient, 6%). Three patients (17%) underwent radiotherapy on brain metastases, while six cases (35%) underwent surgery. Four patients (24%) received a combined strategy with surgery and radiotherapy. Palliative care (steroids alone or in combination with antiepileptics) were adopted in four cases (24%). The description of the therapeutic approach lacked in one case report [21].

Brain metastases occurred in pediatric patients at a median time of 66 (0–96) months from ACC diagnosis, while the first metastatic disease was detected after 60 (0–96) months (Table 2B). Two patients had metastases localized in the lateral ventricle (50%), one in the parietal lobe (25%); in only one patient (25%) the metastases were multiple spreading in the parietal-frontal meninges and the cerebellum. In the remaining five patients the specific anatomical structures were not mentioned. Two patients (40%) referred blurred vision, one (20%) had difficulty in naming and repeating, and in one case (20%) brain metastases caused signs of increased intracranial pressure. In one report brain lesions were asymptomatic (20%), while in four patients no information on symptomatology associated with brain involvement was reported. For the five patients in which the treatment was described, the main approach was surgery (four patients, 80%); in one of them, external ventricular drains and mannitol were necessary to reduce intracranial pressure. Noteworthy, as previously described, brain metastases, as well as skin lesions, resolved spontaneously in one patient, who became disease-free one year after the diagnosis of brain involvement [28].

### Survival outcome

Follow-up data were not available in five patients. In the remaining twenty-two patients, who were fully assessable for prognosis, the median follow-up duration was 38 months (range 3–133 months).

**Fig. 2:**
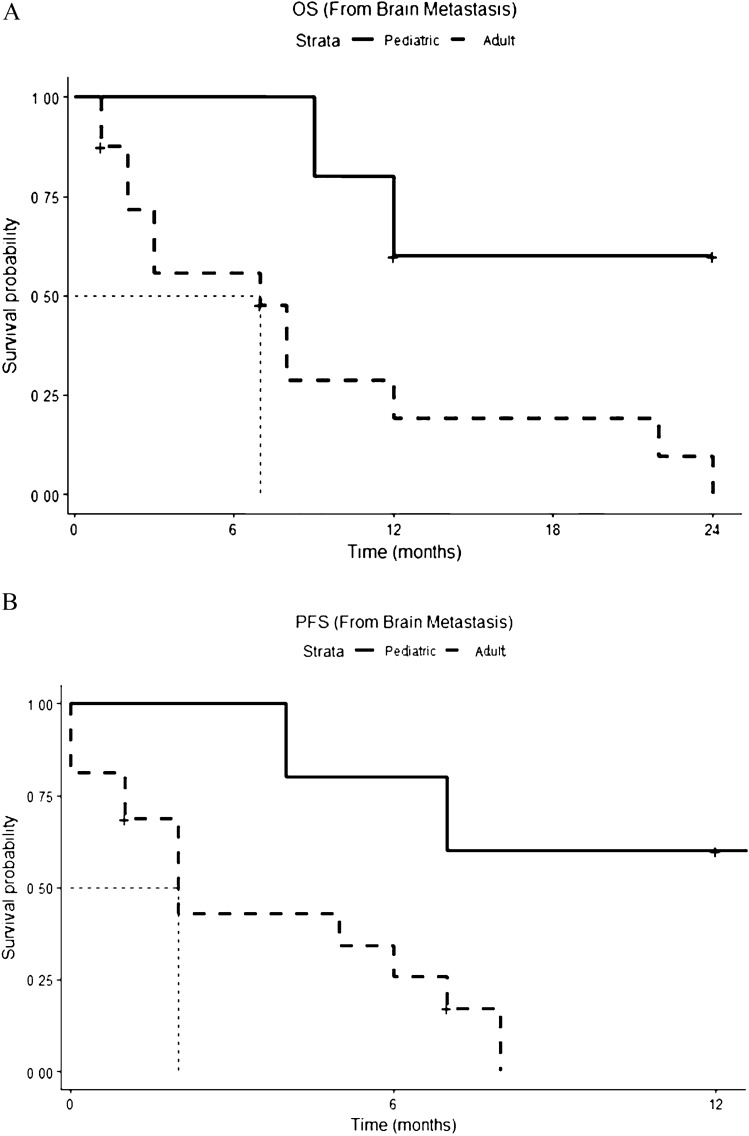
**A**, **B** Overall Survival (OS) (**A**) and Progression Free Survival (PFS) (**B**) from diagnosis of brain metastases: the pediatric population is represented as a continuous line, the adult population as a dotted line

**Fig. 3:**
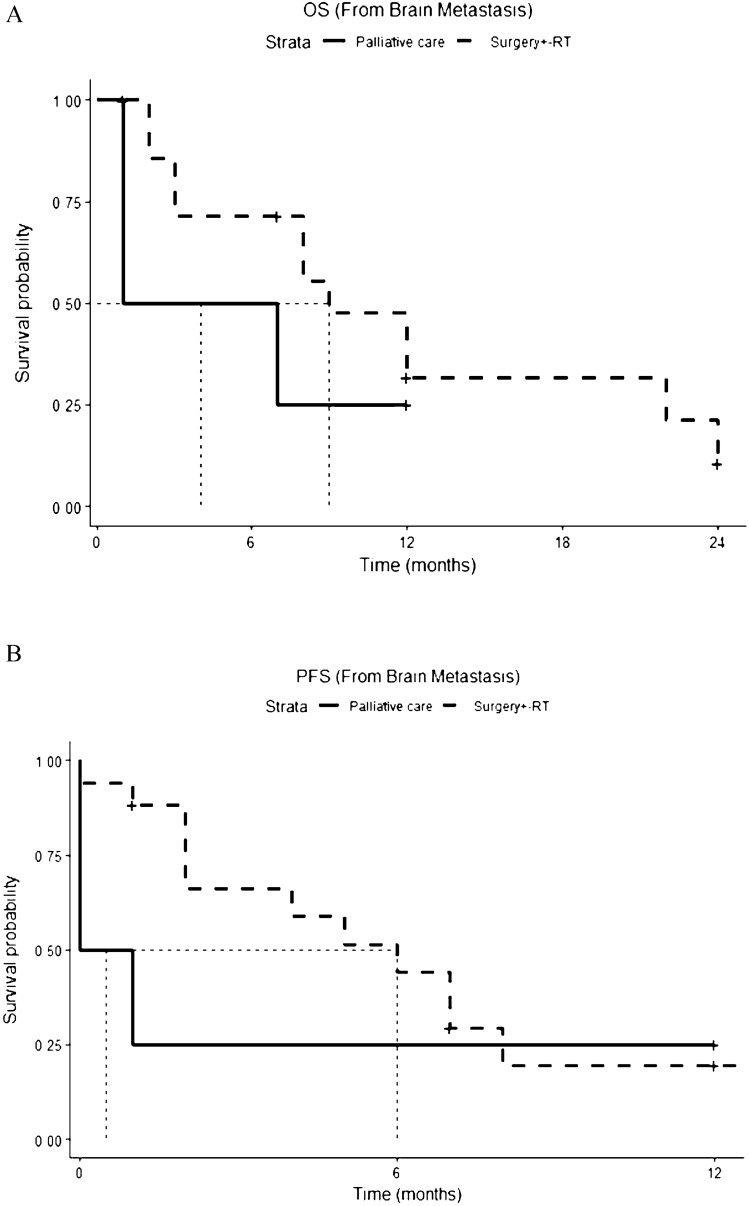
**A**, **B** Overall Survival (OS) (**A**) and Progression Free Survival (PFS) (**B**) from diagnosis of brain metastases in patients treated with radiotherapy ± surgery vs palliative care: palliative care is represented as a continuous line, radiotherapy ± surgery as a dotted line

Data on the disease outcome were available in seventeen adult patients. Among them, three patients died of systemic disease [21, 25, 26] in the absence of intracranial recurrence after local treatment on the brain (surgery alone or surgery followed by radiotherapy); two patients attained the intracranial “no evidence of disease” status (NED) and continued chemotherapy to achieve a systemic control of the disease [26]; twelve patients reported encephalic disease progression [19, 20, 22–24, 26]. Thirteen adult patients died (13/17, 77%). All deaths were disease-related. Median overall survival (OS) and median progression-free survival (PFS) after the diagnosis of brain metastases are shown in Fig. 2A, B, respectively. Median OS from diagnosis of brain metastases was 7 months (1.9–12.0) and median PFS was 2 months (1–3.0). Patients who underwent surgery on brain metastases, either alone or in combination with radiotherapy, had better outcomes, in terms of both OS (Fig. 3A, median 12.0 months vs 5.0 months) and PFS (Fig. 3B, median 8.1 months vs 3.3 months).

Out of the five pediatric patients in whom the outcome was available, four (80%) regained the disease-free status: three patients after surgery (18,20), and one patient after a “wait-and-see” strategy [28]. One of these patients died of metastatic lung disease 3.5 years after the brain disease diagnosis [34]. The remaining patient (20%) experienced intracranial disease progression and died a year after diagnosis of brain involvement [33].

Therefore, two patients (2/5, 40%) in the pediatric population died from disease progression. Median PFS and OS after the diagnosis of brain metastases were not reached (Fig. 2A, B, respectively). As for the adult population, surgery ± radiotherapy on brain metastases had a positive impact on survival, both in terms of OS (Fig. 3A) and PFS (Fig. 3B).

Univariate Cox analysis of the whole series confirmed age (pediatric vs adult) as a positive prognostic factor and leptomeningeal involvement as a negative prognostic factor (Table 3). Patients with leptomeningeal metastases reported a poorer prognosis: median OS was 3.27 months (2.59–5.41) in patients with meningeal involvement vs 11.36 (7.11–15.61) in those without.

**Table 3:** Prognostic factors for Overall Survival according to univariate Cox’s analysis

Clinical characteristics	HR	Univariate 95% CI	P
Age	4.617	1.008–21.141	0.049
Gender	1.042	0.358–3.037	0.940
Functional status	0.768	0.255–2.320	0.640
Cushing’s syndrome	0.700	0.227–2.164	0.536
T at diagnosis	1.565	0.753–3.251	0.230
M at diagnosis	0.977	0.252–3.783	0.973
Adjuvant treatment	1.034	0.502–2.129	0.928
Time interval from diagnosis to brain metastases (months)	0.988	0.970–1.007	0.229
Leptomeningeal involvement	2.435	0.593–9.907	0.030

### Patients and methods

#### Search strategy and case series

A pooled analysis was performed by searching on PubMed the keywords: “brain metastases in adrenocortical carcinoma”, and “leptomeningeal metastases in adrenocortical carcinoma”. We have included both pediatric and adult patients. A manual review of reference lists in relevant publications was carried out to identify additional articles. The last date of the literature search was December 2022. The selection process is shown on the PRISMA flow chart diagram (Fig. 1). Four adult patients followed in our center, Spedali Civili of Brescia, were added to the analysis. These patients were included in the ENSAT registry (www.ensat.org) approved by the Ethical Review Board of ASST-Spedali Civili in Brescia and were treated in accordance with the Declaration of Helsinki. Written consent was obtained from each patient for the recording of pseudonymized and standardized data, including images, in the ENSAT registry for use in any current and future adrenal tumor-related projects.

No PROSPERO registration number was needed.

### Statistical analysis

Data concerning demographics, tumor sizes, histopathological features, and treatments were collected into a database; the resulting population was analyzed as a single cohort.

Survival curves were obtained using the Kaplan–Meier method and compared with the log-rank test.

Exploratory analyses were performed using Cox proportional hazards regression models to test the prognostic value of clinical features and treatment approaches (hazard ratios [HRs] and 95% confidence intervals [CIs]) for overall survival (OS), defined as the time from diagnosis to patient death or the date of the last follow-up, and progression-free survival (PFS), defined as the time from medical or surgical treatment to the progression of disease or death from any cause.

All statistical analyses were obtained using SPSS version 23.0 (SPSS, Chicago, IL) and P values < 0.05 (two-sided) were considered statistically significant.

## Discussion

Brain metastases frequently complicate the natural history of patients bearing lung cancer (36–64%), breast cancer (15–25%), and melanoma (5–20%), but they are rare in ACC patients. Our pooled analysis revealed that only fourteen adult and nine pediatric ACC patients had been described up to now, and we observed 4 patients in our Center out of 225 consecutively observed between 2012 and 2021 (1.8%).

Age at presentation in the present series is in line with the peak of incidence distribution according to age in the adult group, since the median age was 44.5 years (23–63). In the pediatric population, however, the age of onset of brain metastases (9 years) was later than that of the incidence peak. There was a higher prevalence in male patients both in adults and children (56% and 62%, respectively), contrary to what is observed in newly diagnosed ACC patients, in which the female sex prevails [2]. As regards as the onset of brain metastases, we found a noticeable difference in adults versus children: it occurred late in the natural history of the disease in the first setting: 22 months on average from the date of diagnosis of metastatic disease, whereas it was concomitant to the first diagnosis of metastatic disease in pediatric patients. On the basis of these data, brain CT is not mandatory in adult ACC patients at the first diagnosis of metastatic disease. Noteworthy, mitotane, the reference drug of ACC, is notoriously neurotoxic and its neurotoxicity is central, often characterized as attention deficit, disturbed sense of balance, and memory dysfunction [35]. It is sometimes difficult to discriminate neurological symptoms of mitotane toxicity from those of brain metastases. The most frequent symptoms associated with brain metastases from ACC in the present series were hemiparesis and seizures, whereas postural impairment occurred rarely. These findings have potential clinical implications suggesting that the prescription of brain CT or MRI in ACC patients with postural impairments should not be routinely prescribed but be evaluated case by case. When these symptoms appear mitotane levels may be indicative as values above or near the upper limit of therapeutic concentrations (i.e. 20 mg/dl) are more frequently associated with drug-induced neurotoxicity. However, when mitotane levels are in the normal range, neurological symptoms may occasionally occur. A useful suggestion could therefore be to prescribe brain CT or MRI if posture or memory disturbances persist after drug suspension.

In the present series, surgery was performed whenever possible in ACC patients with brain metastases, and this treatment approach, followed or not by radiation therapy, was associated with a better prognosis. Conversely, we were unable to demonstrate the efficacy of systemic therapies in this clinical setting, mainly because brain metastases in adults occurred late in a population already pretreated with standard systemic therapies and the number of pediatric patients with a reported follow-up was limited.

Adult patients had short survival, confirming the concept that brain involvement in ACC patients, as well as in many other oncologic patients, is associated with poor prognosis [36], which could be even worse in the case of leptomeningeal involvement. [37] However, the prognosis of the pediatric population seemed not so poor since the median PFS and OS were not reached, although a higher prevalence of secreting tumors (known as negative prognostic factor). Of note, one child obtained a spontaneous complete remission of brain and skin metastases after surgical removal of primary malignant adrenal disease. This exceptional disease course suggests the possible involvement of the immune system in disease control. The very low number of included children and the limited follow-up could not allow us to make a definitive statement in this respect.

In conclusion, this paper confirms that brain metastases in adult and pediatric patients with ACC are rare. In adults, brain involvement occurs late in the natural history of the disease and is associated with poor prognosis, particularly in the case of leptomeningeal involvement. Children patients with brain metastases seem to have a better prognosis than adults. Surgery plus minus radiation therapy can obtain durable local disease control. Since Temozolomide, the reference drug in the management of primary brain tumors and brain metastases from several malignancies [38, 39], has been demonstrated to be active also in ACC patients [40, 41] it could be a possible systemic option in this clinical setting.

## Conclusion

Brain metastases are extremely rare in patients affected by ACC, worsening their prognosis. Despite their rarity, brain involvement from ACC should not be excluded a priori, especially in presence of neurological disturbs not explainable as toxicities due to mitotane therapy.

### Supplementary Information

Below is the link to the electronic supplementary material.

Supplementary file1 (DOCX 16 KB)

Supplementary file2 (PDF 98 KB)

Supplementary file3 (PDF 57 KB)

Supplementary file4 (PDF 54 KB)

## Data Availability

The raw data supporting the conclusions of this article will be made available by the authors, without undue reservation. All figures presented in this article are original figures, including supplementary figures.
