# Supplementary material for: Outcome of brain metastases from adrenocortical carcinoma: a pooled analysis
Source: J Endocrinol Invest. 2023 Jun 24;47(1):223–34. doi: 10.1007/s40618-023-02140-1 (PMC10776734; doi:10.1007/s40618-023-02140-1)
Supplement: Supplementary file 1 — Supplementary file1 (DOCX 16 KB) [file 40618_2023_2140_MOESM1_ESM.docx]

**Supplementary Table 1. Characteristics of the patients treated at the Medical Oncology Unit of the ASST-Spedali Civili in Brescia**

| **Characteristics** | **Value** |
| --- | --- |
| Patients included in the analysis | 4 |
| Median age (years, range) | 56.5 (42-63) |
| Gender (%)  Male  Female | 3 (75)  1 (25) |
| Ki67 of the primary tumor (%)*  <20%  >=20%  Not available | 0  2 (100)  2 |
| Symptoms at diagnosis (%)  Incidentaloma  Pain  Weight loss  Hormonal secretion | 2 (50)  1 (25)  1 (25)  0 |
| ENSAT stage at diagnosis  II  IV | 2 (50)  2 (50) |
| Time from diagnosis of  primary tumor to brain metastases | 29.5 (2-39) |
| Additional metastases (%)  Lung  Liver  Lymph nodes | 4 (100)  2 (50)  1 (25) |

*The common denominator of the proportions refers to the number of patients for whom the data is available
