# Supplementary material for: Outcome of brain metastases from adrenocortical carcinoma: a pooled analysis
Source: J Endocrinol Invest. 2023 Jun 24;47(1):223–34. doi: 10.1007/s40618-023-02140-1 (PMC10776734; doi:10.1007/s40618-023-02140-1)

**Supplementary Figure 1. Left frontal metastasis at brain MRI in Patient 1 at diagnosis of brain involvement (A, B, C), Brain MRI after surgery in Patient 1 (D, E, F), Disease recurrence in Patient 1 at MRI (G, H, I).**

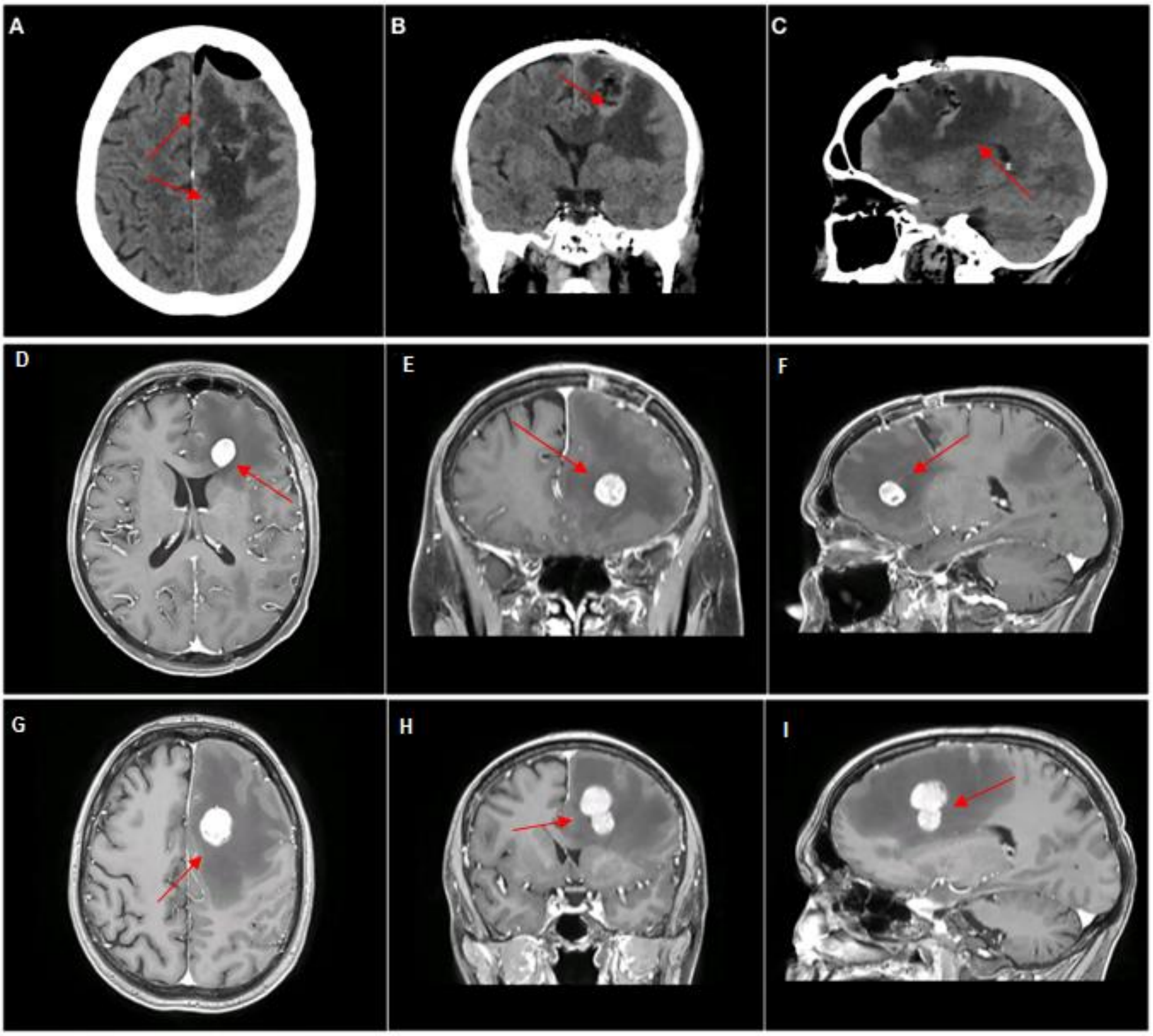

Supplement: Supplementary file 2 — Supplementary file2 (PDF 98 KB) [file 40618_2023_2140_MOESM2_ESM.pdf]
