# Supplementary figures and images for: Outcome of brain metastases from adrenocortical carcinoma: a pooled analysis
Source: J Endocrinol Invest. 2023 Jun 24;47(1):223–34. doi: 10.1007/s40618-023-02140-1 (PMC10776734; doi:10.1007/s40618-023-02140-1)

**Supplementary Figure 2. Brain metastasis and midline shift in Patient 3 at brain CT.**

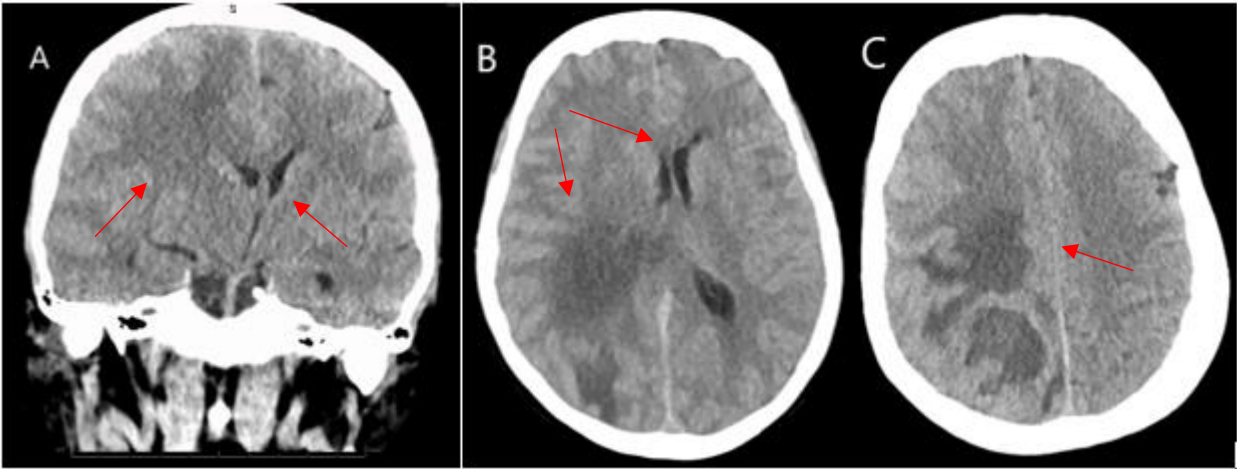

Supplement: Supplementary file 3 — Supplementary file3 (PDF 57 KB) [file 40618_2023_2140_MOESM3_ESM.pdf]

**Supplementary Figure 3. Brain MRI scan in Patient 4.**

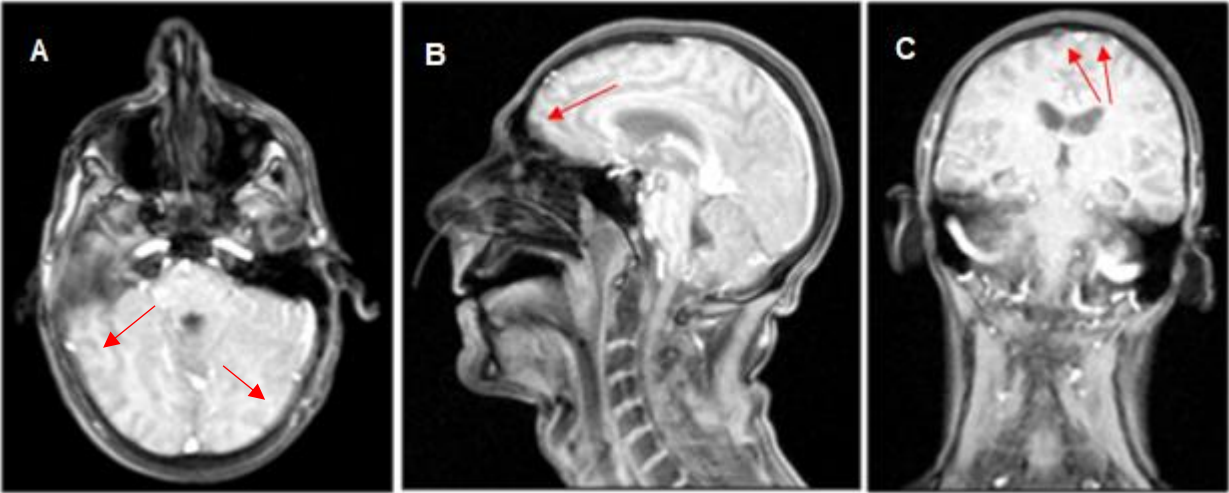

Supplement: Supplementary file 4 — Supplementary file4 (PDF 54 KB) [file 40618_2023_2140_MOESM4_ESM.pdf]
